# Translating aetiological insight into sustainable management of type 2 diabetes

**DOI:** 10.1007/s00125-017-4504-z

**Published:** 2017-11-15

**Authors:** Roy Taylor, Alison C. Barnes

**Affiliations:** 1 Newcastle Magnetic Resonance Centre, Institute for Cellular Medicine, Campus for Ageing and Vitality, Newcastle University, Newcastle upon Tyne, NE4 5PL UK; 2 Human Nutrition Research Centre, Institute of Health and Society, Newcastle University, Newcastle upon Tyne, UK

**Keywords:** Aetiology, Beta cell function, Liver fat, Low-energy diet, Management, Pancreas fat, Pathophysiology, Type 2 diabetes, Weight loss, Weight maintenance

## Abstract

Using a low-energy diet as a tool, it has been possible to elucidate the sequence of pathophysiological changes that lead to the onset of type 2 diabetes. Negative energy balance in type 2 diabetes causes a profound fall in liver fat content resulting in normalisation of hepatic insulin sensitivity within 7 days. As the period of negative energy balance extends and liver fat levels fall to low normal, the rate of export of triacylglycerol from the liver falls. Consequent to this, the raised pancreas fat content falls and in early type 2 diabetes, normal first-phase insulin secretion becomes re-established with normal plasma glucose control. This research, driven by the predictions of the 2008 twin cycle hypothesis, has led to a paradigm shift in understanding. Studying the reversed sequence of pathophysiological changes, the linked abnormalities in liver and pancreas have been revealed. Early type 2 diabetes is a potentially reversible condition. Surprisingly, it was observed that the diet devised as an experimental tool was actually liked by research participants. It was associated neither with hunger nor tiredness in most people, but with rapidly increased wellbeing. A defined period of weight loss followed by carefully planned weight maintenance—the ‘One, Two’ approach—has since been applied in clinical practice. Motivated individuals can reverse their type 2 diabetes and remain normoglycaemic over years. A large study is underway to evaluate the applicability of this general approach to routine primary care practice as a long-term management strategy.

**Electronic supplementary material:**

The online version of this article (10.1007/s00125-017-4504-z) contains a slideset of the figures for download, which is available to authorised users.

## Introduction

A new concept of aetiology of type 2 diabetes resulted from several new research observations in 2006 [1]. Over the past decade the predictions of this twin cycle hypothesis have been tested using as a dynamic tool a low-energy diet [2, 3]. A new, simplified view of the physiological basis of type 2 diabetes has emerged, and this points to a novel approach to management. The nutritional intervention designed to evaluate the physiological basis of the twin cycle hypothesis was observed to be surprisingly well accepted by research participants, and therapeutic use has followed. Weight loss averaging 15% of body weight is reproducibly obtained with potential reversal of type 2 diabetes to a sustained non-diabetic state. Although the effect of weight loss in bringing about resolution of type 2 diabetes is not new, the easily explained pathophysiology enhances engagement. Additionally, the routinely reproducible method of bringing about and sustaining 15% weight loss permits clinical application. This review summarises the new information, bringing together metabolic knowledge of disease mechanisms with details of proven nutritional approaches.

## The new understanding of type 2 diabetes

**Fig. 1:**
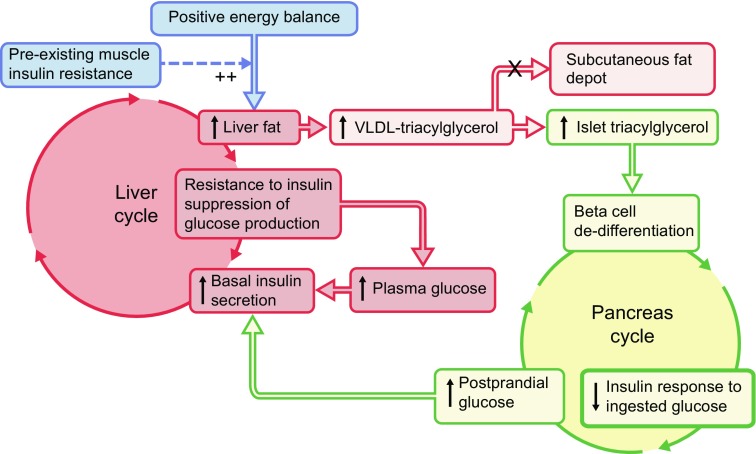
The 2008 twin cycle hypothesis. During chronic intake of more energy than is expended each day, any carbohydrate in excess of requirement must undergo conversion to fat in the liver to permit storage of the metabolic energy as fat [4]. As this process is sensitive to endogenous insulin, individuals with a degree of insulin resistance (and hence higher plasma insulin levels) will tend to accumulate liver fat more readily than others. If the subcutaneous adipose tissue stores have reached capacity, the newly synthesised fat will accumulate in the liver together with excess dietary fat. There it inhibits insulin suppression of glucose production by the liver, and a vicious cycle of hyperinsulinaemia and increased glucose production becomes established. Too much fat in the liver leads to increased export of fat in the form of VLDL-triacylglycerol [5] (shown in lighter red to indicate this is secondary to loss of insulin suppression of liver glucose production). When subcutaneous fat storage capacity is exhausted (i.e. exceeds the personal fat threshold), this will increase fat delivery to all tissues, and the pancreatic islets take up fat avidly [6]. Postprandial hyperglycaemia causes increased and prolonged insulin secretion with further stimulation of de novo lipogenesis. This second vicious cycle thus further increases de novo lipogenesis, and fat delivery to the pancreas. Over many years, the excess pancreas fat brings about loss of specialised function and de-differentiation of the beta cell [7]. Eventually, the inhibitory effects of fatty acids and glucose on the islets reach a trigger level leading to a relatively sudden onset of clinical diabetes. The twin cycle hypothesis predicted that both vicious cycles could be reversed by inducing negative energy balance. Figure adapted from [1] and [8]

The new hypothesis predicted that there were metabolic vicious cycles operating in liver and pancreas, and that these should be able to be reversed. The primary driver of the metabolic problem was identified as positive energy balance, and reversing this was predicted to restore normal blood glucose control (Fig. 1) [1]. Each step shown in Fig. 1 has subsequently been tested and confirmed.

In individuals with a short duration of type 2 diabetes, an average of 15% weight loss allowed fasting plasma glucose to return to normal within 7 days [2]. The rapid return to normal was associated with a 30% decrease in liver fat content and normalisation of liver insulin sensitivity. The COUNTERacting Pancreatic inhibitiOn of INsulin secretion by Triglyceride (Counterpoint) study also demonstrated that over the 8 week study period the level of fat in the pancreas gradually decreased and in step with this, first-phase insulin response returned to within the normal range [2]. In contrast, there was no change in muscle insulin resistance.

**Fig. 2:**
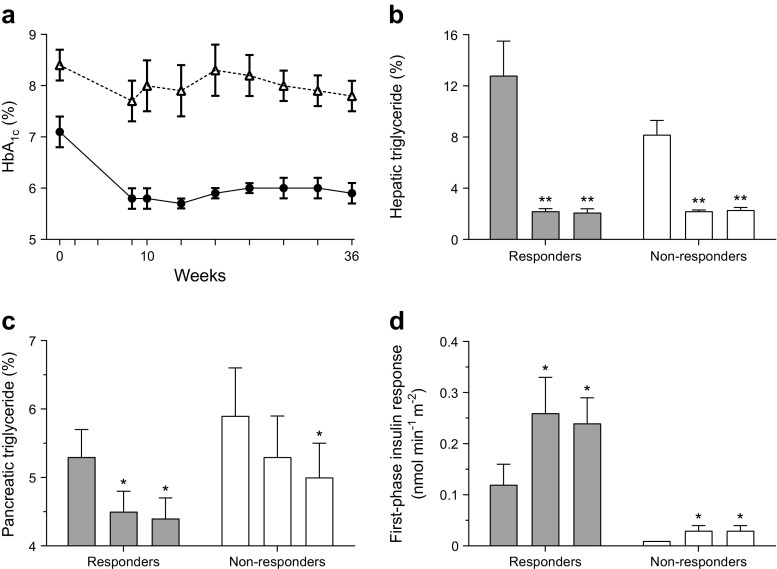
The Counterbalance study. Thirty people with type 2 diabetes of up to 23 years duration lost approximately 15 kg in weight then maintained steady weight for 6 months. Those achieving non-diabetic fasting plasma glucose levels were classified as responders and those whose levels remained in the diabetic range, as non-responders. In (**b**-**d)** the three columns for each group represent (from left to right) baseline on usual drug therapy; after weight loss, on an isoenergetic diet; and after a further 6 months of weight stability. (**a**) The change in HbA_1c_ in responders (circles) and non-responders (triangles). (**b**) There was a similar and sustained decrease in liver fat content in both groups despite ongoing overweight or obesity. (**c**) In the responders, pancreas fat decreased to low levels, but in the non-responders only fell to levels equivalent to baseline responder levels. (**d**) At baseline, the first-phase insulin response was higher in responders and increased to normal levels, whereas the grossly deficient baseline level in non-responders hardly changed. Although non-responders normalise both liver fat content and liver insulin sensitivity, plasma glucose remains elevated in the absence of normalised beta cell function and low fasting plasma insulin levels [9]. *p < 0.05; **p < 0.01 vs baseline. To convert HbA_1c_ values from % to mmol/mol, subtract 2.15 and multiply by 10.929. Triglyceride is elsewhere referred to as triacylglycerol. Copyright: ADA [9]. Copyright and all rights reserved. Material from this publication has been used with the permission of ADA

If the observed changes did represent normalisation of the underlying mechanisms of type 2 diabetes, rather than merely the effect of underfeeding, the beneficial changes should persist after return to normal eating. This was tested in the COUNTERacting BetA cell failure by Long term Action to Normalize Calorie intakE (Counterbalance) study. During a 6 month period of weight stability, levels of fat in the liver and pancreas remained constant, with normal function of these organs in those individuals returned to non-diabetic metabolic control (Fig. 2) [9].

Importantly, after the 15% weight loss in Counterbalance, half of the participants still had a BMI > 30 kg/m^2^, but this had no impact upon their ability to avoid subsequent intra-organ fat re-accumulation. Each individual had dropped below a personal threshold permitting safe storage of fat in the subcutaneous compartment, and this was equally so whether their BMI dropped from 40 to 36 kg/m^2^ or from 28 to 24 kg/m^2^. This illustrates the important concept of the personal fat threshold [10]. The ability to store fat safely in adipose tissue varies considerably between individuals with otherwise normal metabolism [11, 12], and once that capacity has been exceeded the body has to use other storage sites, including liver and pancreas. Individuals can exceed their personal fat threshold at BMIs well within the normal range, as illustrated by the finding that the type 2 diabetes prevalence for women with a BMI of < 22 kg/m^2^ is 4.3-fold that for women with a BMI of 23–24 kg/m^2^ [13]. That is a remarkable increase in relative risk for an increase in BMI that small within the ‘normal’ range. In the United Kingdom Prospective Diabetes Study [14], 36% had a BMI < 25 kg/m^2^. The distribution is right-shifted from that of the UK population of that time, when 64% had a BMI < 25 kg/m^2^ [15]. As the risk of type 2 diabetes rises steeply at higher BMIs, and higher BMIs are now more prevalent, the association between obesity and type 2 diabetes is much more evident today. It is important to recognise BMI as a descriptor for populations (for which the measure was originally developed), rather than a measure to interpret precisely at the level of the individual.

The observation that type 2 diabetes appeared to be a state of excess fat in the pancreas causing the pathognomonic defect in beta cell function, raises the question of how the fat could be interfering with beta cell function. A series of studies has demonstrated that excess saturated fat produces endoplasmic reticulum stress, and that this is associated with de-differentiation of the beta cell [16–19]. It appears to enter a survival mode in which its specialised functions are shut down. Removal of the fat allows re-expression of the insulin gene and resumption of acute glucose-mediated insulin secretion [7].

Understanding what is happening in the pancreas is the key to type 2 diabetes, as the condition never occurs without a major decrease in acute insulin secretion [20, 21]. The volume of the pancreas in type 2 diabetes is only a half to two-thirds that of people with normal glucose tolerance [22, 23]. Also, the pancreas in type 2 diabetes has a markedly irregular border. As a consequence, when selecting a volume of tissue by imaging to be within the pancreas it is more difficult to include only pancreas parenchyma and not the visceral fat that fills in the irregular margin. Additionally, fat within the pancreas comprises both intracellular fat, which is metabolically active, and fat in scattered isolated adipocytes, and imaging methods cannot distinguish between these. Hence, the observed range of fat content is wide in normal and diabetic individuals. This explains the failure of cross-sectional studies to distinguish a difference in total pancreas fat between groups of people with or without type 2 diabetes [24–26]. Induced differences in the metabolically active fraction are evident only in longitudinal studies using precise magnetic resonance technology [2, 3, 9]. After acute weight loss, pancreas fat decreases only in people with type 2 diabetes [3]. Because identification of a tissue volume entirely within the pancreas is difficult, an ‘MR-opsy’ method has been developed to biopsy uniformly sized, small volumes within the pancreas to increase precision and to permit comparison of data between different institutions [27]. The higher the pancreas fat, the lower the pancreas volume, and the nature of this relationship requires further work, perhaps in animal models. The abnormalities of low pancreas volume and irregularity of pancreas border in type 2 diabetes are striking, and further studies are urgently required to understand the relationship between these changes and with both fat content of the organ and aetiology of the condition.

Type 2 diabetes is commonly regarded as being heterogeneous, with possible differing aetiologies in different groups requiring specific approaches to therapy [28]. Individuals vary markedly with respect to any physiological variable, and both genetic and environmental heterogeneity is clear [29]. However, it must be considered that the observed heterogeneity resides in the individuals, not in the basic mechanisms of disease. A parallel could be drawn with observations on the effect of changing an indisputably single mechanism. For example, lisinopril exerts very different effects on blood pressure in different individuals [30]. Observation of the heterogeneity of response to antihypertensive therapy (due to ethnic, genetic and environmental factors affecting each individual) may be interpreted by an observer blinded to the nature of the therapy to suggest that several different drugs are being used. In reality, a single drug is being applied to individuals who respond in a heterogeneous manner. In the same way, the single cause of ectopic fat affecting beta cell function, revealed by the studies discussed above, could be misinterpreted as different specific causes. These considerations apply only to type 2 diabetes itself—a very common condition. There are some clear-cut rare conditions such as monogenic, slow-onset type 1 and pancreatic diabetes that must be considered when making a diagnosis, and there may be other rare conditions that are as yet unrecognised. However, type 2 diabetes itself is a disease so simple that prevalence increases dramatically when a population has ready access to cheap food and decreases when food is scarce [31, 32]. When the Pima Indians were living as subsistence farmers, a comprehensive health survey reported only one case of diabetes, and Joslin famously observed no diabetes at that time [33, 34]. Yet subsequent overnutrition revealed susceptibility to type 2 diabetes in around 40% of the population [35]. Added to this is the steadily increasing beta cell defect as disease duration increases in any one individual, indicating that duration must be taken into account in comparing groups. The lack of precise knowledge of duration of disease in most people adds to apparent heterogeneity. Hence, it is postulated that anyone who develops true type 2 diabetes has accumulated more fat in the pancreas than they individually can tolerate. The cause of this common disease is not heterogeneous, unlike the individuals.

**Fig. 3:**
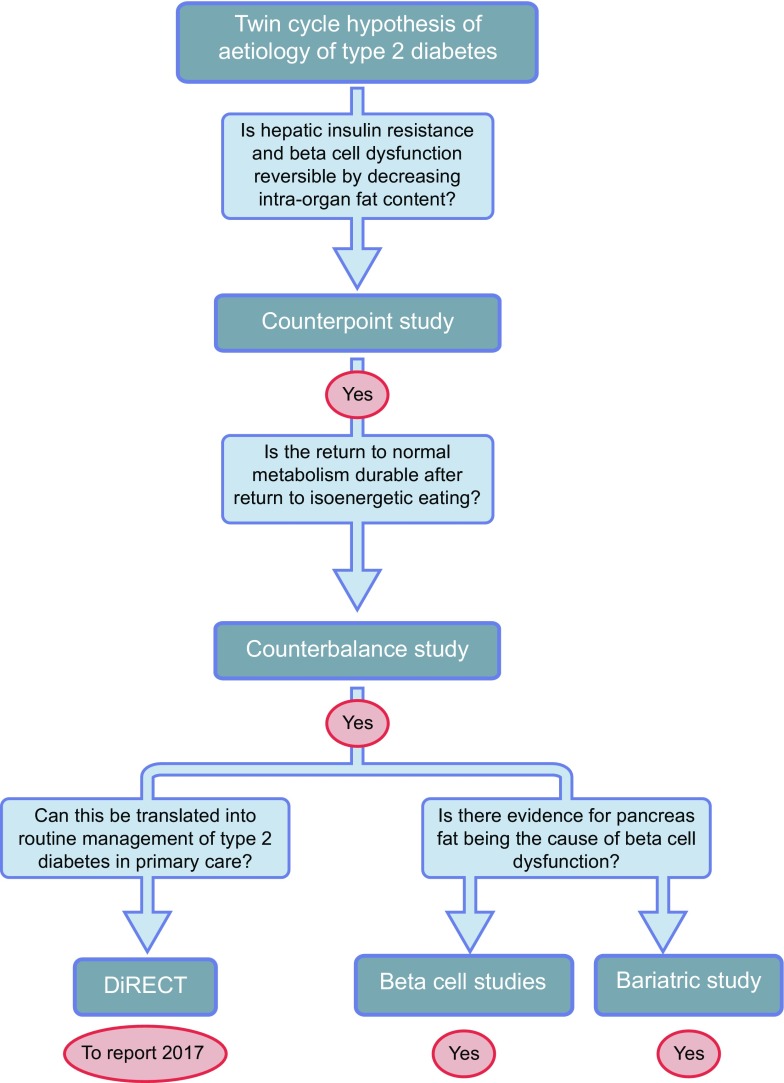
Testing the twin cycle hypothesis. The Counterpoint study established that negative energy balance brought about normalisation of liver fat and insulin sensitivity to suppression of liver glucose production within 7 days, and normalisation of pancreas fat with normalisation of first-phase insulin response over 8 weeks [2]. The Counterbalance study demonstrated that if weight was kept steady after rapid weight loss then the normalisation of liver and pancreas fat content and function was durable during normal, isoenergetic eating [9]. It also showed that reversibility became much less likely after 10 years’ duration of type 2 diabetes. In vitro studies on insulin-producing cells demonstrate that excess fat provision reversibly decreases glucose-stimulated insulin production and that this is due to de-differentiation of beta cells [8, 16, 18]. Studies of acute weight loss produced by bariatric surgery observe a decrease in pancreas fat in people who used to have diabetes and not in non-diabetic individuals [3]. The practicality of achieving long-term reversal of type 2 diabetes in primary care is currently being evaluated in a head-to-head comparison with conventional management [36]

The hypothesis-driven sequence of studies revealing the pathogenesis of type 2 diabetes by observing changes during the return to normal glucose metabolism is illustrated in Fig. 3.

## Testing the twin cycle hypothesis by inducing 15% weight loss

Conventional dietetic approaches to achieving weight loss are not notably successful. The prolonged nature of most interventions is a drawback, and the two major adverse factors associated with this approach are hunger and daily decisions about what and how much to eat. These potentially modifiable factors were accounted for in the design of the Counterpoint and Counterbalance studies with a short, time-limited weight loss phase and a subsequent weight maintenance phase—a ‘One, Two’ approach.

To induce weight loss, a liquid formula diet provided in individual meal sachets was used (2.51 MJ [600 kcal]/day; three sachets). To minimise constipation, up to 240 g/day of non-starchy vegetables were allowed. The total energy intake was thus around 2.93 MJ (700 kcal)/day. A relatively high sugar content was necessary for palatability, but this did not prevent normalisation of fasting plasma glucose within 7 days despite withdrawal of oral hypoglycaemic agents [2]. Unexpectedly, the dietary approach adopted to allow the hypothesis to be tested was actually liked by the participants.

Counterbalance demonstrated that the individuals who did not return to non-diabetic blood glucose control typically had a longer disease duration, but notably already had severely impaired beta cell function at baseline [9]. Weight loss produced normalisation of liver fat content with normalisation of hepatic insulin sensitivity in all, but this alone was insufficient to normalise glucose control. The apparent heterogeneity of response lay in the duration-dependent progression to complete beta cell de-differentiation, whereby those individuals with beta cells still at the reversible stages could achieve complete re-differentiation and resumption of beta cell specialist function [7].

All participants reported almost complete lack of hunger within 1–2 days of commencing the diet. This is striking during achievement of ~ 15 kg weight loss. Notable wellbeing was reported [37]. Several participants wished to continue the low-energy liquid diet after the 8 week period of the study to meet their own weight target. A recent systematic review exposes the misconception that rapid weight gain inevitably follows rapid weight loss [38]. If weight loss by any regimen is not followed up by a supportive programme it is unlikely to be sustained. In Counterbalance, participants were seen at monthly intervals for the 6 month follow-up period and weight remained stable. Importantly, they were advised in advance to expect to be eating only two-thirds of their usual intake of food. The concept that weight loss is more difficult to achieve in people with type 2 diabetes is incorrect [38].

Following media interest in the Counterpoint results, a large number of emails and letters were received from people with diabetes, and full how-to-do-it information was placed on the Magnetic Resonance Centre website [39]. Analysis of the initial email responses to the information showed that half had used a liquid diet replacement, and half had used small portions of ordinary foods [40]. Weight loss was approximately 15 kg in both groups, illustrating the practical possibility of achieving this degree of weight loss by any energy-restricted approach that is sustainable. There was clearly an important matter of individual preference.

## Nature of the ongoing support required after reversal of diabetes

The liquid low-energy diet is prescriptive, rendering it reassuringly straightforward to follow. In contrast, the prospect of returning to normal eating after the liquid diet, with inherent decisions about what and how much to eat, is often a time of great anxiety. RCT evidence confirms that a gradual transition to the weight maintenance diet following weight loss with a low- or very-low-energy diet over a period of weeks is associated with improved weight maintenance at 12 months [41, 42]. A gradual, stepwise change from the low-energy liquid diet to normal foodstuffs was employed in the subsequent studies. During this phase of Counterbalance, weekly face-to-face review was undertaken, with directive advice on what and how much to eat, consideration being given to individual dietary preferences and calculated energy requirements. The energy prescription for weight stabilisation was estimated from achieved body weight using predictive equations [43]. Additional telephone support was also available. Monthly review and weekly self-weighing was then undertaken [44], resulting in weight stability over 6 months. One critical ingredient for success was observed to be family support. If the spouse or partner was not supportive, then the chance of long-term success was low. Notably, in both Counterpoint and Counterbalance spouses/partners reported losing weight as well. Changing the obesogenic microenvironment of the home is critical.

**Fig. 4:**
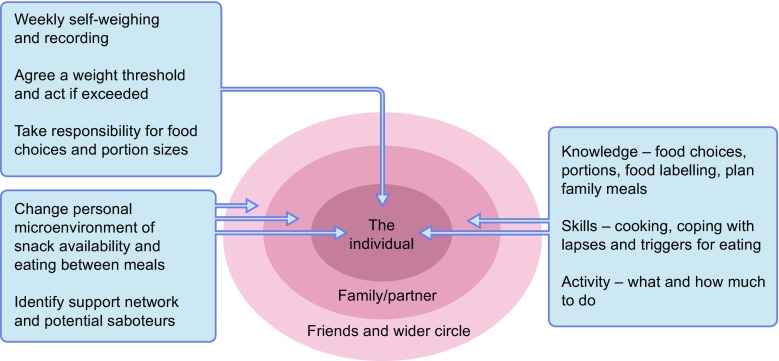
Components of support to provide long-term weight stability. The major components of support for the individual are indicated. As eating is a social activity, the wider context must be taken into account. Maintenance of support from family/a partner is critical, and understanding from friends and work colleagues is also key, along with coping strategies where such support is absent. All of these points require exploration at ongoing follow-up visits. Not illustrated in the diagram is input of outside agencies as required and the crucial potential for the whole obesogenic environment to be influenced by policy makers

While some individuals can successfully maintain a new lower body weight under their own direction [40], most are likely to require ongoing support to limit weight regain over time [45]. The support that may be required during weight loss maintenance is summarised in Fig. 4.

## Nature of ongoing diet

An individual who has achieved remission of type 2 diabetes through weight loss remains inherently predisposed to recurrence if there is a return to chronic energy excess. It is therefore of utmost importance to maintain the body weight at a level below the personal fat threshold. However, long-term maintenance of lost weight is recognised as the most challenging aspect of obesity treatment [46]. With appropriate support, significant weight loss can be maintained over the longer term [47].

So which dietary strategy is most appropriate? A low-fat diet (< 30% total energy from fat) has long been the mainstay of dietary advice. This is traceable to the association of high fat intake with cardiovascular death reported in the seven countries study [48]. Epidemiological associations from cross-sectional studies have been shown repeatedly not to represent cause and effect [49], but the belief is ingrained in practice and reflected in current guidelines for type 2 diabetes. However, it is now well established that it is adherence to any strategy rather than a particular macronutrient composition which is likely to result in long-term weight maintenance. Over recent years nutrition guidelines have moved away from prescriptive macronutrient content towards a more person-centred philosophy, acknowledging that there is no ‘one best diet’ for diabetes [50]. Three evidenced approaches (low carbohydrate, Mediterranean and intermittent energy restriction) are discussed in the following section as potential options for a maintenance diet following the initial rapid weight loss period.

For many Europeans, decreasing or omitting the carbohydrate portion of the main meal, with or without change in other meals, is a simple change, easily achievable in the context of family eating. Low-carbohydrate diets (less than 120 g per day of total carbohydrate) and very-low-carbohydrate diets (20–50 g total carbohydrate per day) for diabetes management continue to excite vehement debate [49, 51]. A carbohydrate-restricted diet implies an increase in the ratio of fat to carbohydrate, conflicting with long-held beliefs about the risks of higher-fat diets. There is insufficient evidence to recommend an ideal proportion of total energy from carbohydrate in diabetes management [52]. For equivalent weight loss, different compositions of diet do not affect liver fat content nor any other aspect of fat distribution [53]. Despite incorporation into evidence-based nutrition guidelines in 2011 [50], UK clinical practice relating to carbohydrate restriction has not kept pace with the evidence base [54]. Low-carbohydrate diets have been reported to be superior to low-fat diets for short to medium term use in type 2 diabetes, with comparable longer-term (> 12 months) outcomes [55].

The Mediterranean diet is based on a combination of foodstuffs high in monounsaturated fats (primarily olive oil), legumes and vegetables, and with restricted red meat, processed foods and refined carbohydrates. Such diets consistently give benefit in terms of weight control and cardiovascular health [56–58]. A decrease in diabetes incidence independent of weight has also been observed [59]. Given the current evidence, the Mediterranean diet could be recommended as an option for a weight maintenance diet regardless of diabetes remission status. A combined Mediterranean and carbohydrate-restricted diet may be particularly beneficial for those for whom weight loss has not resulted in diabetes remission, almost halving the need for diabetes drugs over 4 years following diagnosis of type 2 diabetes [60].

Novel time-limited approaches to eating (alternate day or intermittent fasting, time-restricted eating and meal timing during the day) provide an alternative to daily energy restriction. Intermittent energy restriction achieved by a variety of methods is as effective as daily energy restriction in achieving weight loss and maintenance for up to 12 months [61]. Weight loss outcomes were better in studies that applied an energy prescription on the non-fasting (‘feed’) days in order to ensure an overall weekly deficit. In some studies mean weight losses were similar between groups but the proportion of participants losing a clinically significant amount of weight (≥ 5%) was higher with intermittent energy reduction (60–65% at 12 weeks) compared with the daily energy restriction group (37%) [62]. One day of energy restriction per week maintained the improvements in weight and insulin sensitivity (achieved using a 5:2 approach) over 6 months of follow-up in overweight or obese women. The first published results using the 5:2 approach in a population with type 2 diabetes demonstrate comparable reductions in weight and HbA_1c_ between daily and intermittent energy-restricted groups with no adverse effects on exercise levels or appetite [63, 64]. Evidence relating to the use of this approach for longer-term weight maintenance is lacking and warrants further study.

Studies reporting an association of omission of breakfast with increased weight usually have a clear commercial bias [65]. Prospective study reveals no disbenefit of omitting breakfast in terms of eating more later in the day and demonstrates the potential major energy advantage [66]. For some individuals omission of breakfast may usefully contribute to long-term weight control if they do not like breakfast and can readily extend the overnight fast to lunchtime [67].

As different dietary approaches are likely to suit the needs and preferences of different individuals (especially those who have had little success with conventional low-fat diets), a decision aid such as that developed to facilitate informed choices about diabetes medications may be a useful tool. Long-term weight control will be most effectively achieved by finding an approach to eating that an individual, and their family, can sustain. The composition (restricted carbohydrate/restricted fat) is far less important than the overall quantity of food, and there are varying, equally valid ways of achieving this goal.

## Physical activity

There is clear-cut evidence for encouraging sustainable, daily physical activity during the long-term weight maintenance phase, alongside energy control [68]. This is of great importance. However, little attention has been paid to the compensatory eating phenomenon, which is a major hazard of the weight loss phase itself [69–71]. If overweight or obese individuals embark upon an exercise regimen, there tends to be an increase in weight due to an increase in energy intake, partly conscious and partly subconscious. This varies between individuals [70], but extra exercise can be avoided with no disbenefit to substantial weight loss. The energy expenditure during exercise that is tolerated by most overweight, older people is modest and easily cancelled out by a snack. During the weight loss phase of both Counterpoint and Counterbalance, participants were asked to continue usual activities but to avoid any increase in physical activity. This appeared to contribute to the achievement of the target ~ 15% weight loss in 8 weeks. In contrast, the more widely used approach of advising an increase in exercise from the beginning of a longer-term period of a diet with modest energy restriction may contribute to falling short of the weight loss target [72]. A randomised study testing the effect of avoiding additional prescribed exercise during the weight loss phase of any energy-restricted diet may be helpful in clarifying the effectiveness of this approach.

## Previous studies of weight loss as management for type 2 diabetes

Observation of the beneficial effect of weight loss on blood glucose control is not new. Ayurvedic scriptures from 2000 years ago recognised fat and thin forms of diabetes and recommended fasting and exercise [73]. Bouchardat described the resolution of glycosuria in diabetes during the famine caused by the 1870 siege of Paris [74]. Allen’s low-energy diet also permitted prolonged survival of individuals with what is now recognised as type 1 diabetes [75]. Energy restriction brought about by bariatric surgery has long been known to return blood glucose control to normal in some people [76]. A 6 day very-low-energy diet supervised in hospital was shown to improve fasting plasma glucose from 18.6 to 11.2 mmol/l, but in common with all such studies did not consider the effect of long duration of diabetes [77]. Two studies are notable in using a low-energy diet with follow-up over 1 year [78] and 5 years [79] to demonstrate the beneficial effect on glucose control. In the latter study, one out of 15 remained normoglycaemic off all medications, giving a very favourable ‘number needed to treat’ for the complete reversal of diabetes.

The only older study to have used a very-low-energy diet as a tool to evaluate mechanisms of disease was published in 1986 [80]. In-patient supervision of a 1.38–2.51 MJ/day (330–600 kcal/day) diet for up to 40 days was followed by 60–380 days of outpatient follow-up. This study specifically avoided additional exercise in the weight loss phase, and is one of the few in which participants achieved weight loss of around 15%. This decrease caused a fall in fasting plasma glucose from 15.4 to 6.8 mmol/l, which correlated with a fall in hepatic glucose output. However, the study group of eight included people with a long duration of diabetes, and no change was observed in average insulin response to oral glucose.

## Lifelong reversal of type 2 diabetes?

Information from observational studies on large groups of people with type 2 diabetes suggest an inexorable worsening of blood glucose control with a need for increasing numbers of tablets and, eventually, insulin [81, 82]. The associated demonstration of a steady decline in beta cell function [83] has given rise to the widespread belief that this is inevitable. However, these observational studies were carried out only in the situation of maintained or increasing body weight [84].

The demonstration that the non-diabetic control of glucose metabolism is fully maintained over 6 months is useful in confirming the mechanistic basis of long-term remission of type 2 diabetes [9]. In those who had achieved a post-weight-loss fasting plasma glucose of < 7 mmol/l, liver and pancreas fat content fell to normal levels. First-phase insulin response became and remained normal. There was no accumulation of fat in either pancreas or liver, even though mean BMI was 30 kg/m^2^ [9]. These data are important in clarifying the physiological basis of continuing reversal of type 2 diabetes, separate from the well-recognised human problem of avoiding weight regain in the face of unchanged environmental pressures. Longer-term data are required from formal studies, but non-diabetic metabolic control can be maintained over several years, providing that weight regain is avoided [72, 79, 85].

The Diabetes Remission Clinical Trial (DiRECT) is a large randomised trial that will determine the effectiveness of rapid weight loss followed by supportive follow-up in primary care, head-to-head with conventional management [36]. It will report 12 month outcomes at the end of 2017. The intervention consists of 8 h of training for the practice nurse (or practice dietitian if available), with continuing specialist dietitian support. All oral glucose-lowering agents are withdrawn. The dietary and behavioural approach known as CounterWeight has been trialled for obesity management in primary care with 1 year follow-up demonstrating maintenance of ≥ 15 kg weight loss in 30% [47]. DiRECT will address the question of selection bias and degree of self-motivation that follows from the smaller, pathophysiological studies. It will also provide further evidence of the basic mechanisms of disease. Rapid recruitment to DiRECT reflects the extreme dislike of type 2 diabetes by many people. This is generally underestimated by professionals and, given the previous lack of means to restore normal metabolism, has led to a situation of learned helplessness. The outcomes of DiRECT may indicate an important way forward.
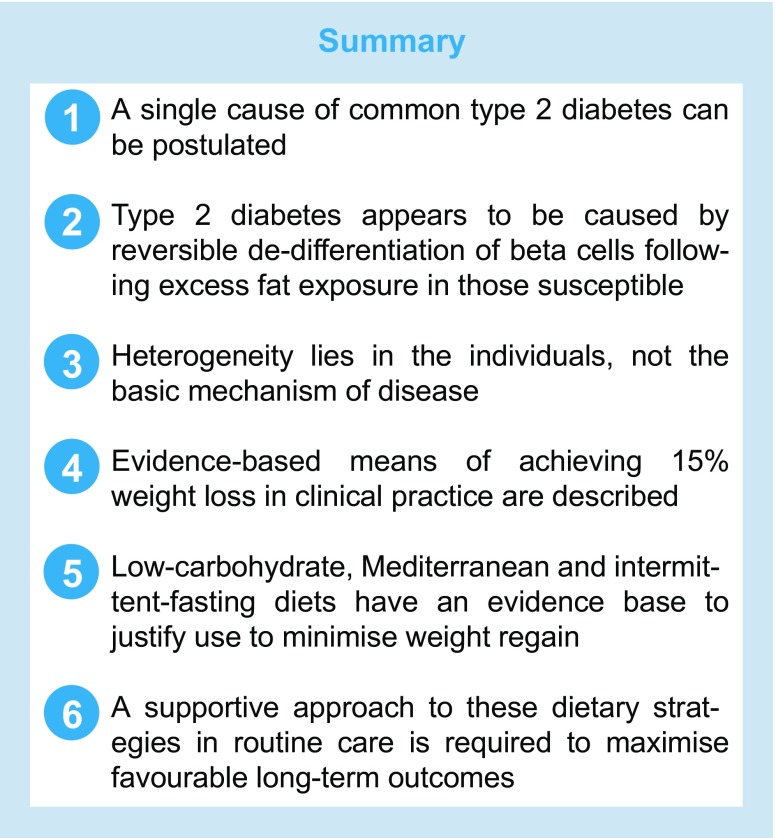


## Electronic supplementary material

ESM (PPTX 506 kb)

## Abbreviations

Counterpoint: COUNTERacting Pancreatic inhibitiOn of INsulin secretion by Triglyceride
Counterbalance: COUNTERacting BetA cell failure by Long term Action to Normalize Calorie intakE
DiRECT: Diabetes Remission Clinical Trial
