# Supplementary material for: Translating aetiological insight into sustainable management of type 2 diabetes
Source: Diabetologia. 2017 Nov 15;61(2):273–83. doi: 10.1007/s00125-017-4504-z (PMC6448962; doi:10.1007/s00125-017-4504-z)
Supplement: Supplementary file 1 — (PPTX 506 kb) [file 125_2017_4504_MOESM1_ESM.pptx]

## Slide 1
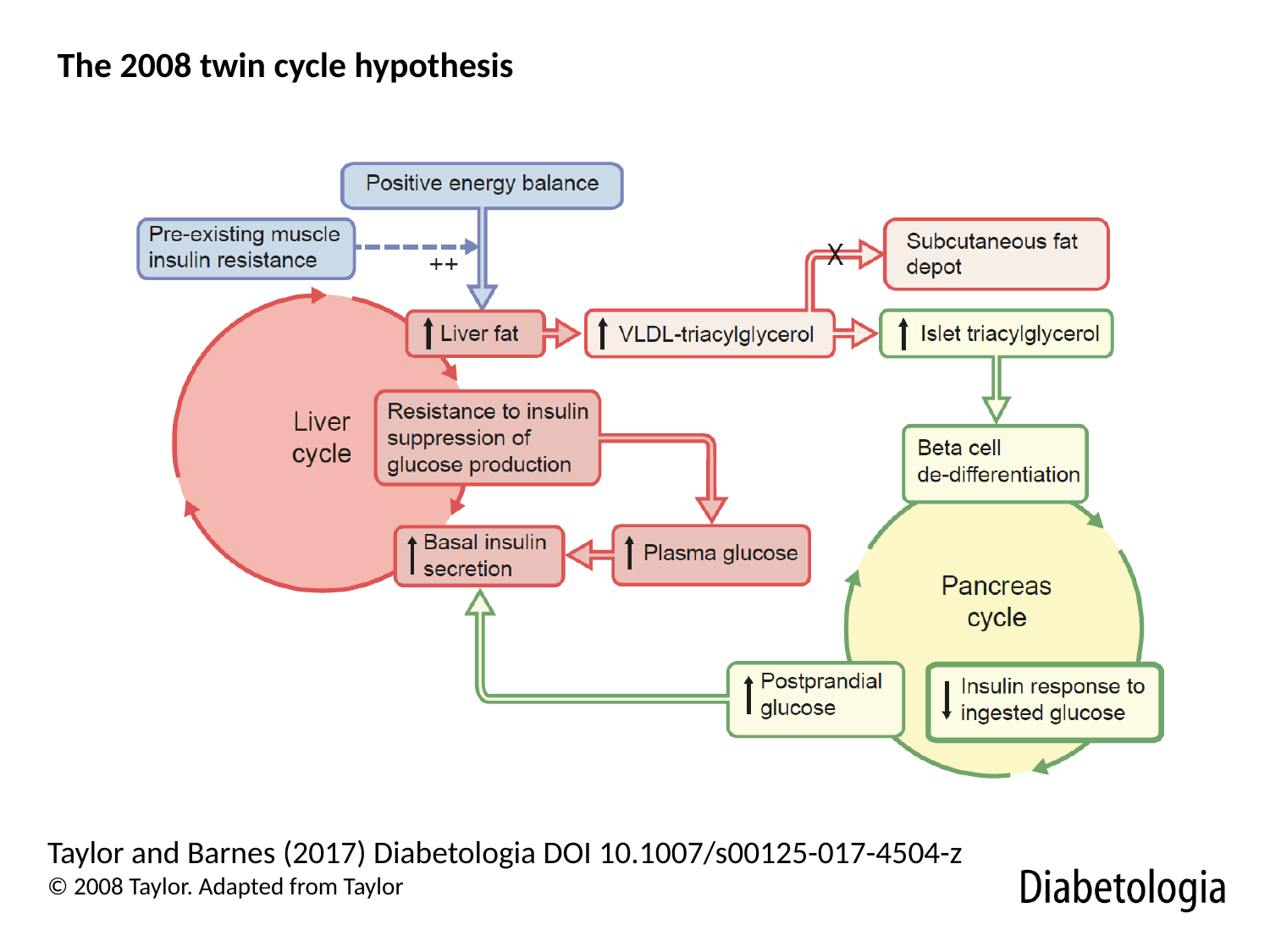

The 2008 twin cycle hypothesis
Taylor and Barnes (2017) Diabetologia DOI 10.1007/s00125-017-4504-z
© 2008 Taylor. Adapted from Taylor

## Slide 2
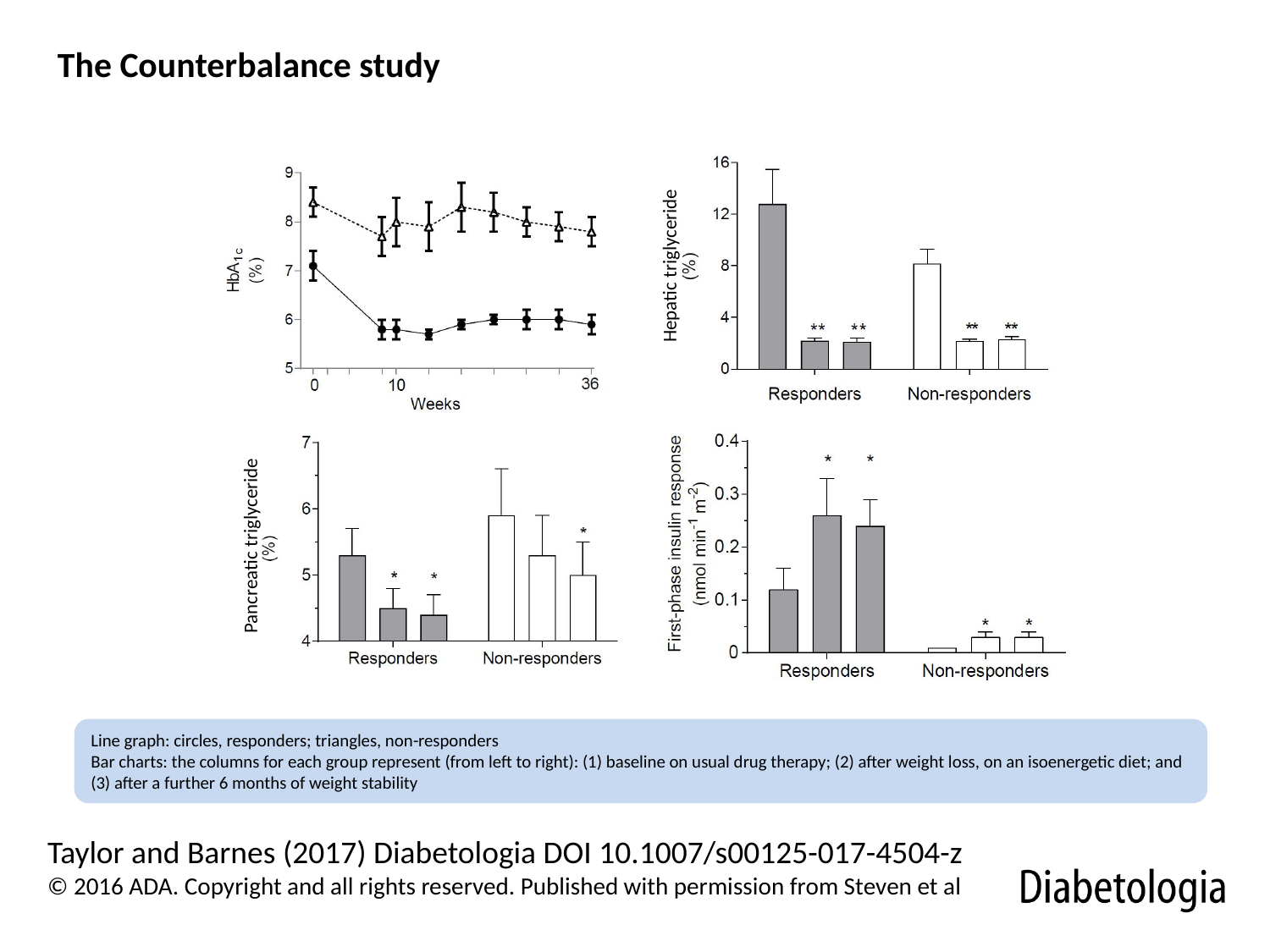

The Counterbalance study
Hepatic triglyceride
Pancreatic triglyceride
Line graph: circles, responders; triangles, non-responders
Bar charts: the columns for each group represent (from left to right): (1) baseline on usual drug therapy; (2) after weight loss, on an isoenergetic diet; and (3) after a further 6 months of weight stability
Taylor and Barnes (2017) Diabetologia DOI 10.1007/s00125-017-4504-z
© 2016 ADA. Copyright and all rights reserved. Published with permission from Steven et al

## Slide 3
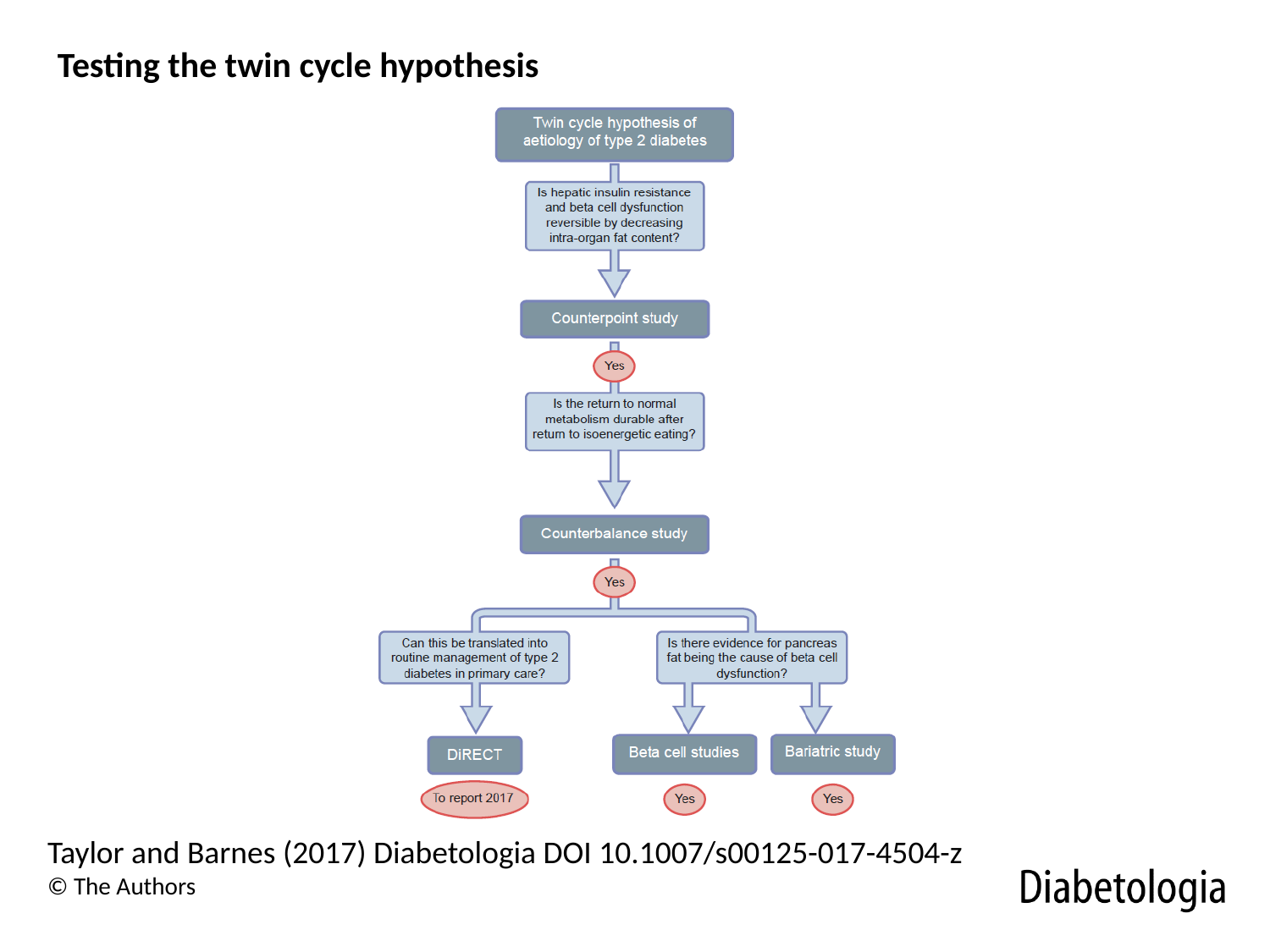

Testing the twin cycle hypothesis
Taylor and Barnes (2017) Diabetologia DOI 10.1007/s00125-017-4504-z
© The Authors

## Slide 4
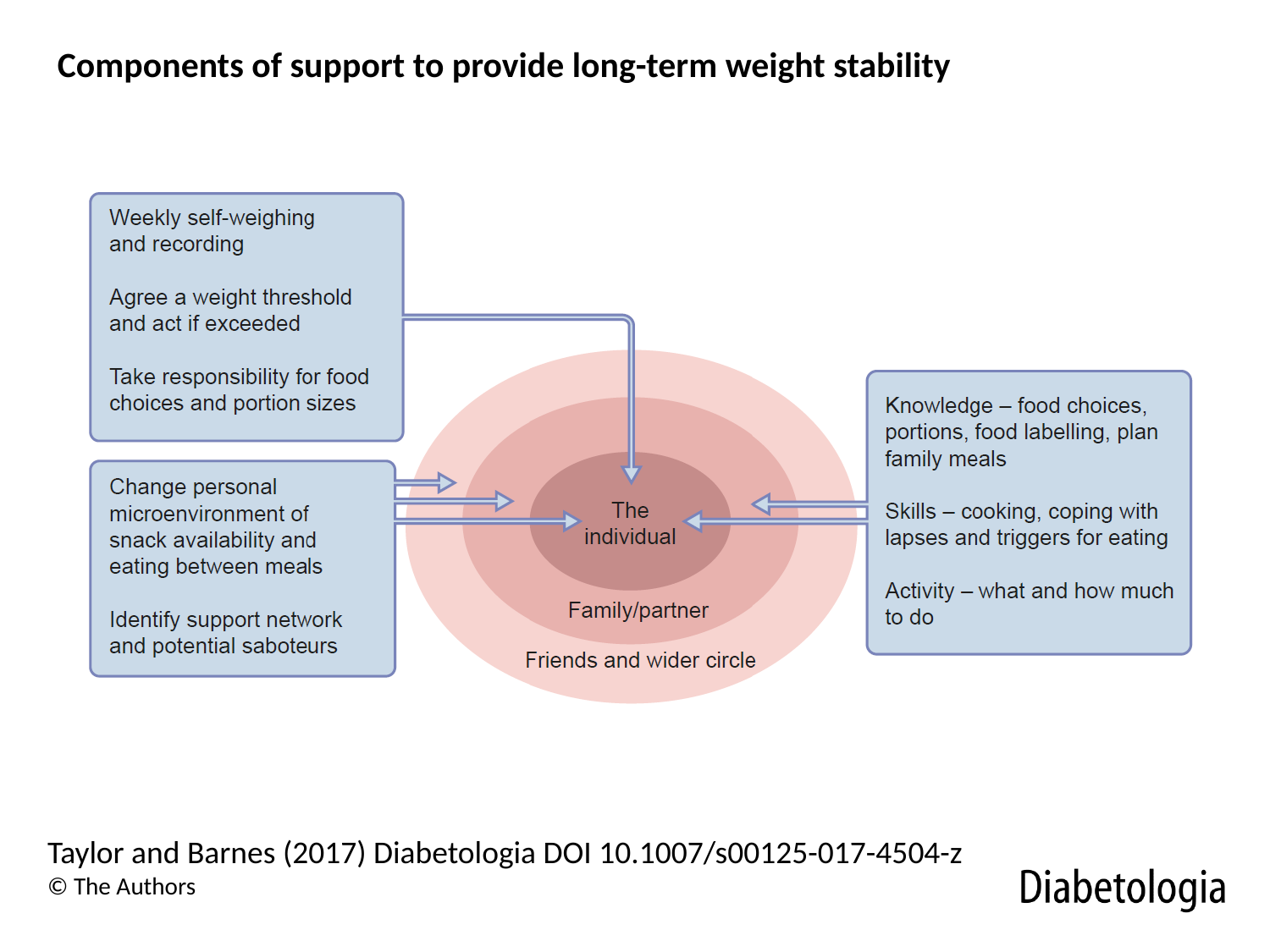

Components of support to provide long-term weight stability
Taylor and Barnes (2017) Diabetologia DOI 10.1007/s00125-017-4504-z
© The Authors
